# Neutrophil-to-lymphocyte ratio as an early marker of outcomes in patients with advanced non-small-cell lung cancer treated with nivolumab

**DOI:** 10.1007/s10147-018-1250-2

**Published:** 2018-02-13

**Authors:** Aya Nakaya, Takayasu Kurata, Hiroshige Yoshioka, Yuki Takeyasu, Maiko Niki, Kayoko Kibata, Naoko Satsutani, Makoto Ogata, Takayuki Miyara, Shosaku Nomura

**Affiliations:** First Department of Internal Medicine, Kansai Medical University, 2-5-1, Shin-machi, Hirakata, Osaka 573-1010 Japan

**Keywords:** Neutrophil-to-lymphocyte ratio (NLR), Nivolumab, Non-small-cell lung cancer (NSCLC), Progression-free survival (PFS), Immune-related adverse event (irAE)

## Abstract

**Background:**

There is an unmet need to identify markers that predict the response to nivolumab in patients with non-small-cell lung cancer (NSCLC). The neutrophil-to-lymphocyte ratio (NLR) was recently recognized as an indicator of a poor prognosis in patients with various cancers. In the present study, we quantified the predictive impact of NLR in patients with NSCLC treated with nivolumab.

**Methods:**

We retrospectively analyzed 101 patients with advanced NSCLC treated with nivolumab at Kansai Medical University Hospital from December 2015 to December 2016. Patients were administered nivolumab at a dose of 3 mg/kg every 2 weeks. The predictive value of NLR for disease progression before treatment and 2 and 4 weeks after nivolumab treatment was assessed.

**Results:**

The median progression-free survival (PFS) of patients with an NLR of < 3 before treatment was 3.4 months, whereas that of patients with an NLR of ≥ 3 was 2.9 months (*p* = 0.484). The median PFS of patients with an NLR of < 3 at 2 weeks after treatment was 5.3 months, whereas that of patients with an NLR of ≥ 3 was 2.1 months (*p* = 0.00528). The median PFS of patients with an NLR of < 3 at 4 weeks after treatment was 5.3 months, whereas that of patients with an NLR of ≥ 3 was 2.0 months (*p* = 0.00515).

**Conclusion:**

The NLR at 2 and 4 weeks after treatment might be a useful marker for the prediction of the treatment response or disease progression in patients with advanced NSCLC receiving nivolumab.

## Introduction

Nivolumab is the first immune checkpoint inhibitor to be approved for relapse and refractory non-small-cell lung cancer (NSCLC) and has been the mainstay of treatment for NSCLC since 2015. Pembrolizumab was recently approved for first-line treatment of NSCLC. Thus, the advent of immune checkpoint inhibitors has significantly changed the treatment of NSCLC. When using pembrolizumab, a programmed death ligand 1 (PD-L1) expression level of > 50% is essential [1]. However, it has been reported that whether the PD-L1 expression level over 1, 5, and 10% or not is related with the efficacy of nivolumab in patients with NSCLC [2, 3]. No reliable biomarker for prediction of the clinical outcome after nivolumab treatment has yet been established in daily clinical practice. Nivolumab is only effective in a portion of patients with NSCLC, and it is very expensive. Identifying biomarkers that predict treatment efficacy and thereby allow appropriate patient selection for these treatments is a crucial topic of ongoing research.

Inflammation is widely recognized to play an integral role in both the development and propagation of various cancers. Increased systemic inflammation is associated with a poorer prognosis in patients with cancer. The neutrophil-to-lymphocyte ratio (NLR), calculated as the absolute neutrophil count divided by the absolute lymphocyte count within the peripheral blood, has been shown to correlate with the prognosis of various malignancies [4–7]. The NLR has been evaluated in patients with both localized and advanced NSCLC and appears to be prognostic in these patient populations [8–10]. Furthermore, several reports have indicated that the NLR might predict the effects of cancer immune checkpoint inhibitors in patients with melanoma [11, 12].

The purpose of the present study was to determine whether the NLR is associated with the response to nivolumab therapy in patients previously treated for advanced NSCLC.

### Patients and methods

We conducted a retrospective study of patients with NSCLC who were treated with single-agent nivolumab after platinum failure from January 2015 to December 2016 at Kansai Medical University Hospital, Osaka, Japan. Eligible patients had NSCLC with a history of platinum failure. Previously untreated patients were excluded from the study. Nivolumab (3 mg/m^2^) was administered every 2 weeks until the occurrence of disease progression, unacceptable toxicity, withdrawal, or death. NLR was evaluated before treatment and 2 and 4 weeks after the first treatment. We categorized the patients into two groups according to an NLR cut-off value of 3. This cutoff was previously validated as being associated with inferior overall survival (OS) in patients with lung cancer in the largest such study to date [13]. This study was conducted in accordance with the Declaration of Helsinki and the requirements of the institution’s review board.

### Study assessments

Patient treatment responses were evaluated according to the Response Evaluation Criteria in Solid Tumors using whole-body computed tomography performed every 8–12 weeks. However, because of the possibility of pseudo-progression in patients considered to have progressive disease, tumor size was carefully evaluated with reference to the guidelines for the Evaluation of Immune Therapy Activity in Solid Tumors [14]. The evaluation of toxicity was based on the Common Toxicity Criteria for Adverse Events, version 4.0 [15]. Adverse events (AEs) were evaluated for 12 weeks. Immune-related AEs (irAEs) were defined as rash, diarrhea, colitis, thyroid disorder, hepatitis, arthritis, and other conditions [16].

### Statistical analysis

Progression-free survival (PFS) was defined as the time from the start of single-agent nivolumab treatment to objective disease progression. OS was calculated from the start of single-agent nivolumab treatment until the time of death or the last clinical follow-up. Survival curves were generated using the Kaplan–Meier method, and differences were evaluated using the log-rank test. Multivariate Cox-proportional hazards models were used to determine whether the patient baseline characteristics and/or NLR were associated with PFS. All statistical tests were two-sided. Statistical significance was defined as *p* < 0.05, and 95% confidence intervals were calculated. All statistical analyses were performed using EZR (Saitama Medical Center, Jichi Medical University, Saitama, Japan), which is a graphical user interface for R version 2.13.0 (The R Foundation) [17]. Specifically, EZR is a modified version of R Commander (version 1.6–3), which adds statistical functions frequently used in biostatistics.

## Results

### Patients

The clinical characteristics of the patients (*n* = 101; median age, 69 years, range 45–84 years; 77% male) included in this study are shown in Table 1. Patients were diagnosed with either squamous cell carcinoma (37%) or non-squamous cell carcinoma (63%). Among all patients with adenocarcinoma, 10% had an epidermal growth factor receptor mutation and 3% had the echinoderm microtubule-associated protein-like 4–anaplastic lymphoma kinase (EML4-ALK**)** fusion gene. One, two, and more than three previous regimens had been administered to 18, 28, and 55% of patients, respectively. The median follow-up period was 8.9 months.

**Table 1:** Patients’ characteristics

No. of patients	101
Median age (y/o), range	69 (45–84)
Male sex (%)	77
ECOG PS(%)	
0–1	84
2 ≤	16
Smoking history (%)	
Yes	84
No	16
Histology (%)	
Squamous	37
Non-squamous	63
Targetable driver mutation (%)	
EGFR	10
ALK	3
No. of prior systemic Tx (%)	
1	18
2	28
3 ≤	55

*EGOG* Eastern Cooperative Oncology Group, *PS* performance status, *EGFR* epidermal growth factor receptor, *ALK* anaplastic lymphoma kinase, *Tx* treatment

### Treatment response and survival

**Fig. 1:**
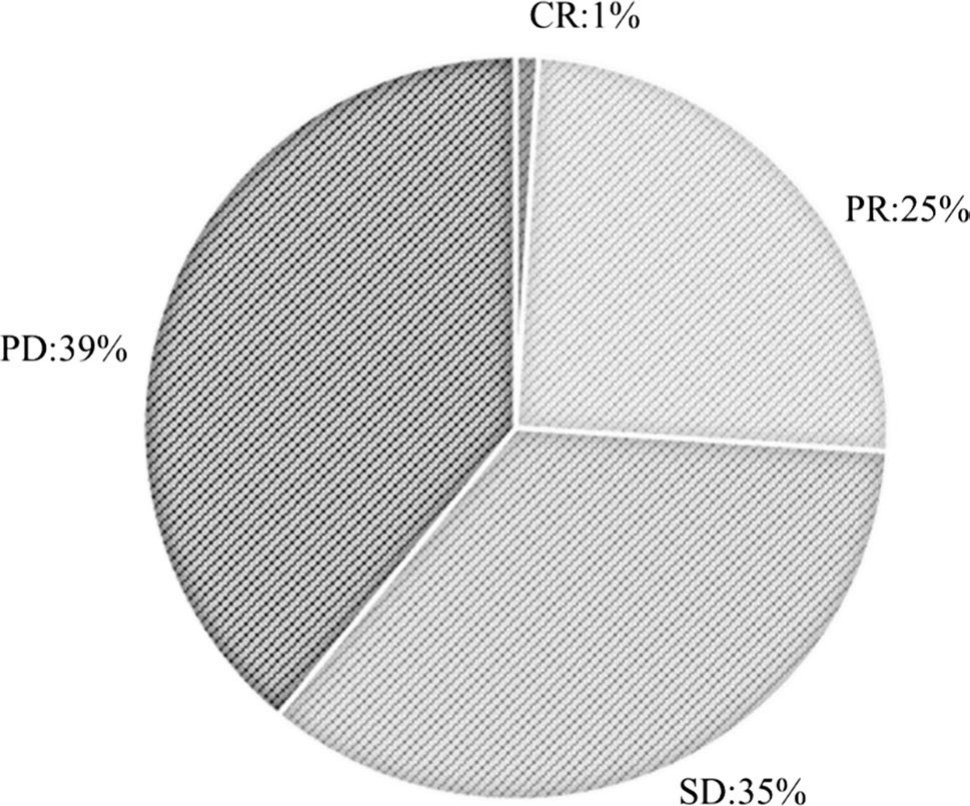
Patients’ treatment response rates. The patients’ treatment responses were as follows: 1%, complete response; 25%, partial response; 35%, stable disease; and 39%, progressive disease

**Fig. 2:**
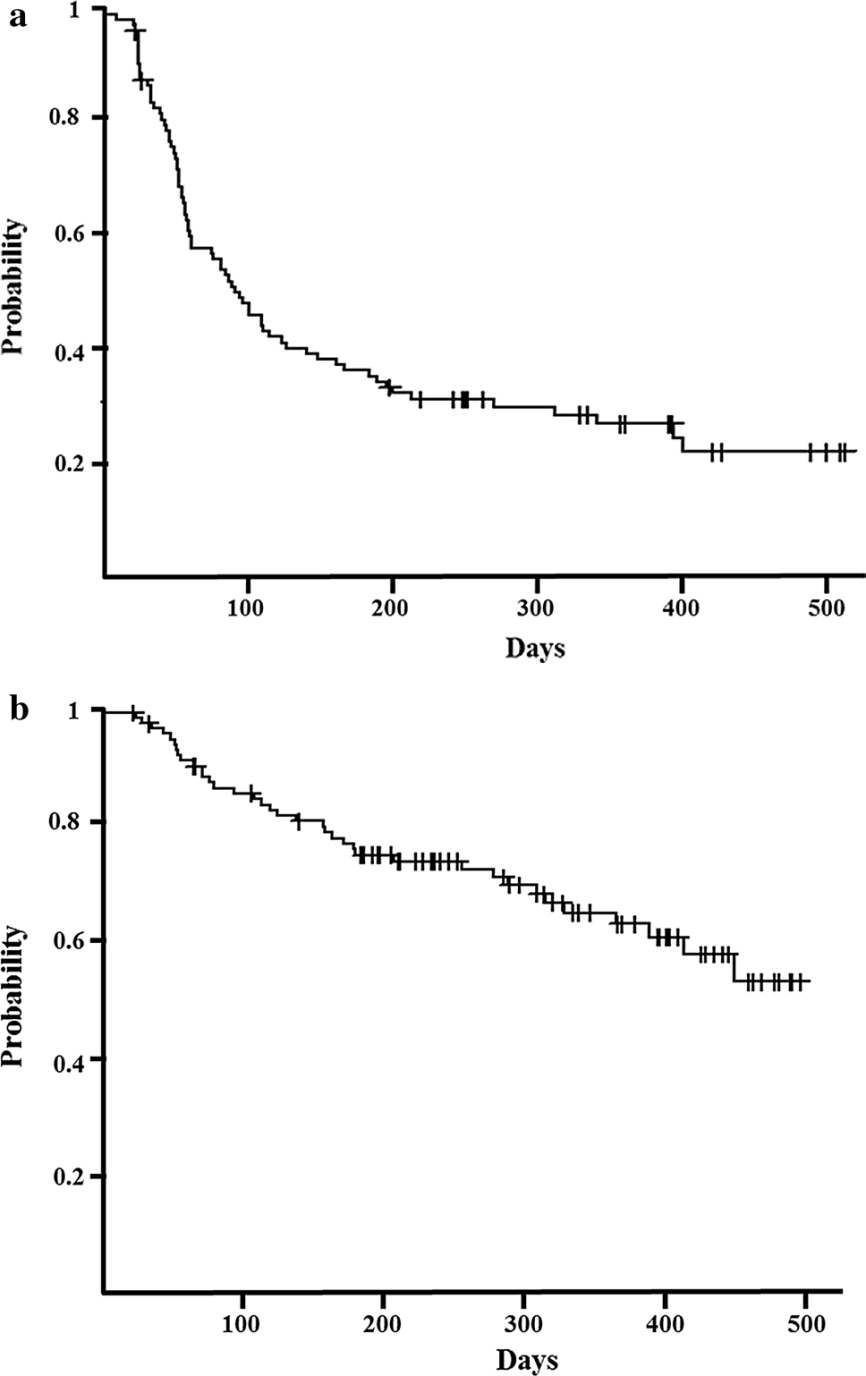
Kaplan–Meier survival analysis. **a** Median progression-free survival, 3.2 months (range 2.1–4.1 months). **b** Median overall survival, 17.0 months (range 12.4 months–not available)

One percent of patients achieved a complete response, 25% achieved a partial response, 35% had stable disease, and 39% had progressive disease (Fig. 1). Median PFS and OS were 3.2 (Fig. 2a) and 17.0 months (Fig. 2b), respectively.

### Relationship between NLR and PFS

**Fig. 3:**
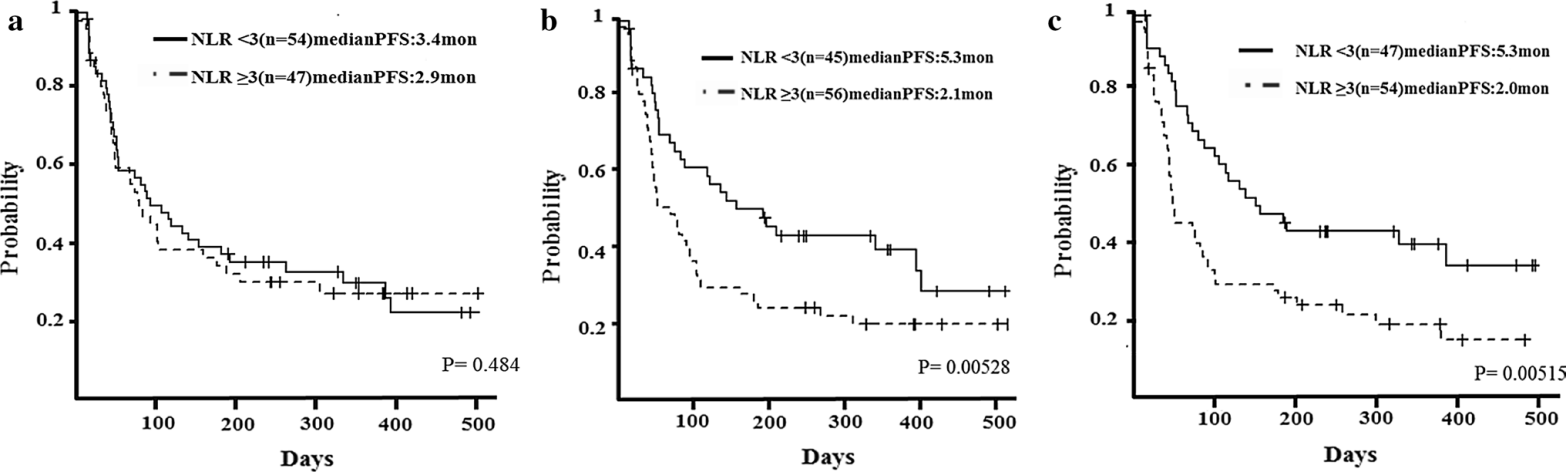
Relationship between neutrophil-to-lymphocyte ratio (NLR) and progression-free survival (PFS). **a** Median PFS of patients with an NLR of < 3 before treatment was 3.4 months, whereas that of patients with an NLR of ≥ 3 was 2.9 months (*p* = 0.484). **b** Median PFS of patients with an NLR of < 3 at 2 weeks after the initial treatment was 5.3 months, whereas that of patients with an NLR of ≥ 3 was 2.1 months (*p* = 0.00528). **c** Median PFS of patients with an NLR of < 3 at 4 weeks after the initial treatment was 5.3 months, whereas that of patients with an NLR of ≥ 3 was 2.0 months (*p* = 0.00515)

We evaluated NLR before treatment and 2 and 4 weeks after treatment to determine its association with PFS. The median PFS of patients with an NLR of < 3 before treatment was 3.4 months, whereas that of patients with an NLR of ≥ 3 was 2.9 months (*p* = 0.484) (Fig. 3a). The median PFS of patients with an NLR of < 3 at 2 weeks after initial treatment was 5.3 months, whereas that of patients with an NLR of ≥ 3 was 2.1 months (*p* = 0.00528) (Fig. 3b). The median PFS of patients with an NLR of < 3 at 4 weeks after initial treatment was 5.3 months, whereas that of patients with an NLR of ≥ 3 was 2.0 months (*p* = 0.00515) (Fig. 3c). We tried to clarify the relationship between the number of prior chemotherapy regimens and NLR. Although with patients with 1–2 prior lines, NLR did not reveal a strong predictive power for PFS (before treatment/2 weeks after initial treatment/4 weeks after initial treatment: *p* = 0.49/0.291/0.0875). For patients with ≥ 3 prior lines, NLR was statistically a useful tool to predict PFS (before/2 weeks/4 weeks: *p* = 0.478/0.00225/0.0104) (data not shown).

### Frequency of AEs

The frequencies of AEs and irAEs are shown in Tables 2 and 3. The most common AEs (any grade) were fatigue (*n* = 26), appetite loss/nausea (*n* = 17), and rash (*n* = 17). Severe AEs (grade > 3) were anemia (*n* = 4), liver dysfunction (*n* = 2), and thyroid disorder (*n* = 2). The most common irAEs were rash (*n* = 15), liver dysfunction (*n* = 2), and thyroid dysfunction (*n* = 2).

**Table 2:** Adverse events

Adverse events	Any grade (*n*)	Grade 3–4 (*n*)
Fatigue	26	1
Appetite loss/nausea	17	0
Rash	15	1
Anemia	9	4
Liver dysfunction	8	2
Thyroid disorder	8	2
Interstitial lung disease	6	1
Edema	5	0
Diarrhea	4	0
Mucositis	4	1
Renal dysfunction	3	0
Fever	3	0
Others	17	8

**Table 3:** Immune-related adverse events

Immune-related adverse events	Any grade (*n*)
Skin	
Rash	15
Hepatic	
Liver dysfunction	8
Endocrine	
Thyroid disorder	8
Adrenal disorder	2
Gastro-intestinal	
Diarrhea	4
Mucositis	4
Pulmonary	
Interstitial lung disease	6
Renal	
Renal dysfunction	3
Others	
Hypoalbuminemia	2
Edema	5
Thrombocytopenia	1
Pleural effusion	1
Parotitis	1

**Fig. 4:**
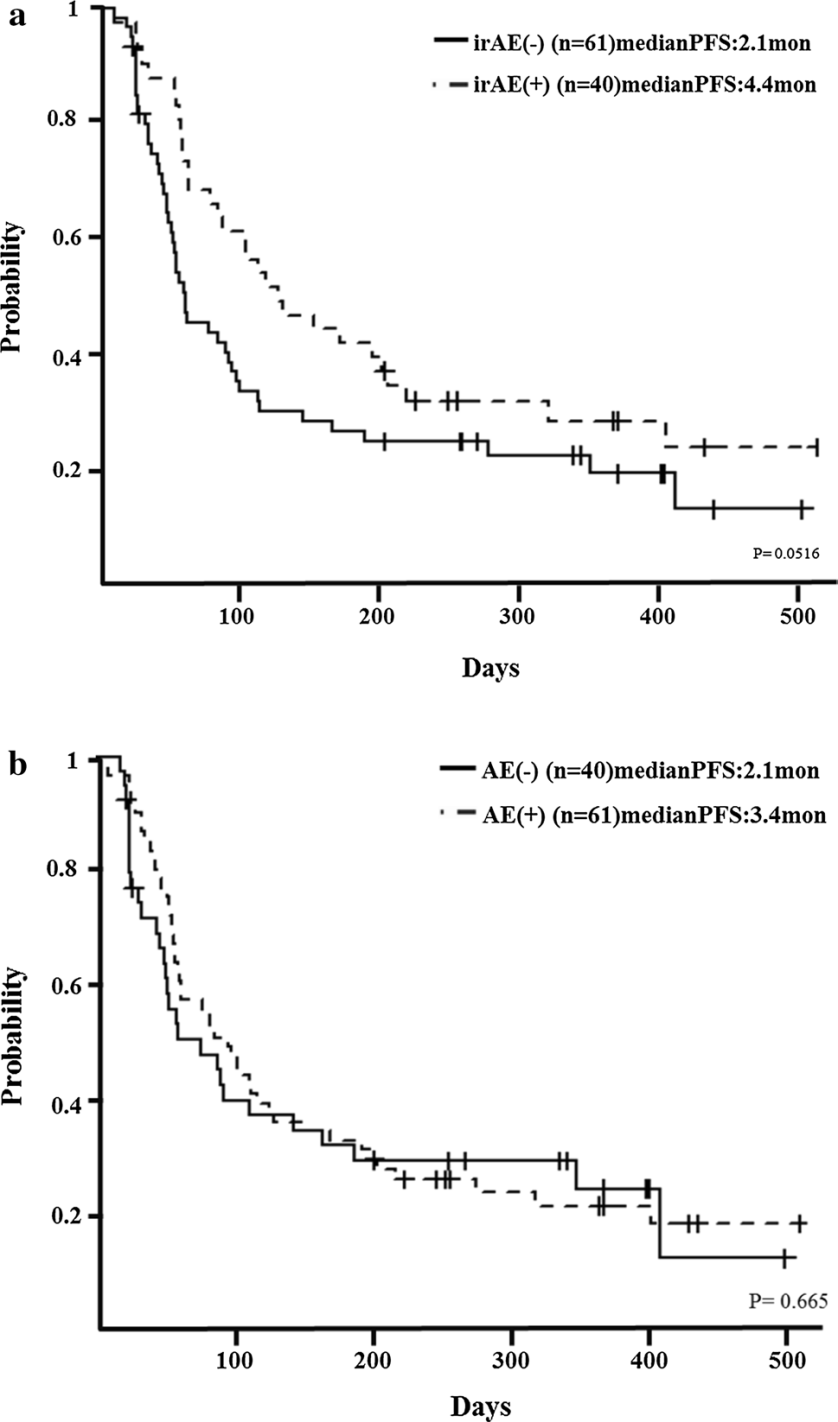
Relationship between adverse events (AEs) and progression-free survival (PFS). **a** Median PFS of patients with immune-related AEs was 4.4 months, whereas that of patients without immune-related AEs was 2.1 months (*p* = 0.0516). **b** Median PFS of patients with AEs was 2.1 months, whereas that of patients without AEs was 3.4 months (*p* = 0.665)

Patients with irAEs (*n* = 40) had better PFS than patients without irAEs (*n* = 61), although the difference was not statistically significant (median, 4.4 vs. 2.1 months, respectively; *p* = 0.0516) (Fig. 4a). Likewise, PFS between patients with AEs (*n* = 61) and without AEs (*n* = 40) was not significantly different (median, 2.1 vs. 3.4 months, respectively; *p* = 0.665) (Fig. 4b).

### Multivariate analysis

We also investigated other factors that may be associated with the outcome of nivolumab treatment, such as age of > 75 years, sex, number of prior chemotherapy treatments, histology, elevation of C-reactive protein, irAEs, and NLR at 2 and 4 weeks after treatment. Multivariate analysis showed that more than three lines of chemotherapy were associated with significantly worse PFS. In contrast, patients with irAEs had significantly better PFS. An NLR of < 3 at neither 2 nor 4 weeks after treatment was associated with significantly better PFS in the multivariate analysis (Table 4).

**Table 4:** Multivariate analysis

	Odds ratio	95%CI	*p*
Age			
≥ 75 years	0.691	0.3876–1.2320	0.2104
< 75 years	1		
Sex			
Male	0.6541	0.3820–1.1200	0.1218
Female	1		
Prior line			
3 ≤	2.012	1.2220–3.3130	0.006019
1–2	1		
Histology			
Squamous	1.36	0.8401–2.2000	0.2111
Non-squamous	1		
C-reactive protein			
≥ 1	0.973	0.5521–1.7150	0.9246
< 1	1		
irAEs			
+	0.5785	0.3404–0.9831	0.04309
–	1		
NLR 2 weeks			
≥ 3	1.368	0.7600–2.4630	0.2959
< 3	1		
NLR 4 weeks			
≥ 3	1.595	0.9278–2.7430	0.0912
< 3	1		

*irAE* immune-related adverse event, *NLR* neutrophil-to-lymphocyte ratio

## Discussion

Our study revealed that an NLR of < 3 at 2 and 4 weeks after nivolumab treatment might be an independent prognostic biomarker in patients in patients with advanced NSCLC. This is the first study to evaluate the power of NLR to predict the outcome of nivolumab treatment in an Asian cohort. Bagley et al. recently found that pretreatment NLR was associated with inferior OS and PFS in patients with lung cancer treated with nivolumab [18]. However, these patients had been treated with several regimens; thus, pretreatment NLR is presumed to have been affected by the previous treatment. In the present study, pretreatment NLR was not associated with PFS.

In recent years, it has become increasingly apparent that cancer-associated inflammation is a key determinant of disease progression and survival in patients with many types of solid tumors [19]. Because immune checkpoint inhibitors enhance antitumor immunity by blocking negative regulators of T-cell function [20], it is plausible that alterations in the relative proportions of circulating lymphocytes could influence the efficacy of immune checkpoint inhibitors. Inflammatory cytokines and chemokines can be produced by both the tumor and associated host cells such as leukocytes, contributing to malignant progression [21]. As an indicator of inflammation and tumor immune response, NLR plays a pivotal role in various cancers. However, the exact mechanism that explains the poor survival outcomes of patients with cancer who have a high NLR remains unclear.

Neutrophils can produce various chemokines and cytokines and suppress the immune activity of lymphocytes and natural killer cells. Tumor-associated neutrophils are derived from peripheral neutrophils and have been proposed as key mediators in tumor progression specifically because they induce genetic instability, promote tumor growth, stimulate angiogenesis, and favor the invasive behavior of cancer cells [22].

The importance of lymphocytes has been highlighted in several studies in which increased infiltration of lymphocytes into tumors was associated with a better response to cytotoxic treatment and progression in patients with cancer [23–25]. Lymphocytes play a crucial role in host cell-mediated immune regulation, which is important for destruction of residual malignant cells [26]. It is widely believed that tumor-infiltrating lymphocytes (TILs) are associated with better clinical outcomes in patients with cancer [27].

The expression of PD-L1 and the presence of TILs in melanoma influence the therapeutic effect of immune checkpoint inhibitors [28]. Positivity of both PD-L1 and TILs is the most effective status for efficacy of immune checkpoint inhibitors. We therefore assumed that NLR might reflect the presence of TILs and predict the therapeutic effect.

We clarified the relationship between the number of prior chemotherapy regimens and NLR. Our result revealed that NLR at 2 and 4 weeks after the initial treatment was a useful predictive factor for PFS using nivolumab in patients with ≥ 3 prior lines, which are known to be related to poor outcome.

Although the mechanism is unknown, NLR reflects the balance between inflammation and immunoreaction and has long been used as an effective prognostic biomarker in patients with various cancers, including lung cancer [18, 29–31].

Interestingly, irAE-positive patients had better PFS in the present study. In a previous study, irAE-positive patients with melanoma treated with nivolumab had better OS [27]. This is the first study to evaluate the relationship between irAEs and PFS in patients with NSCLC treated with nivolumab. IrAEs might be a prospective factor of nivolumab efficacy in patients with NSCLC.

This study has a few limitations. First, the most effective cut-off value of the NLR is unknown. In our cohort, the median NLR was 2.6. We considered whether to set a cutoff of 2 or 3. With a cutoff of 2 or more, the number of patients over the cutoff of 2 is about 70% and there is a bias. On the other hand, with a cutoff of 3 and above, balance of the number of patients below and above the cutoff is about fifty–fifty. The median PFS of patients with an NLR of < 2 before treatment (*n* = 27) was 5.0 months, whereas that of patients with an NLR of ≥ 2 was 2.9 months (*n* = 71) (*p* = 0.258). The median PFS of patients with an NLR of < 2 at 2 weeks after the initial treatment(n = 16) was 7.0 months, whereas that of patients with an NLR of ≥ 2 (*n* = 82) was 2.9 months (*p* = 0.347). The median PFS of patients with an NLR of < 2 at 4 weeks after the initial treatment (*n* = 19) was 7.1 months, whereas that of patients with an NLR of ≥ 2 (*n* = 19) was 2.7 months (*p* = 0.024). There was no statistically significant difference with a cutoff of 2. Therefore, we adopted a cutoff of 3 in the current study. Furthermore, we used a cutoff of 3 according to the largest cohort study performed to date [13]. However, various studies of the usefulness of NLR have used different cut-off values, and the methods of selecting these NLR cutoffs were unclear in many studies. Prospective studies are needed to verify the adaptive cut-off value. Second, NLR at 2 and 4 weeks after the initial treatment was significantly associated with PFS; however, we failed to identify a pretreatment biomarker. Because nivolumab is an expensive agent, an effective biomarker must be identified before we decide to use this agent. Although irAEs are an efficient biomarker, they cannot serve as a pretreatment biomarker. Thus, another biomarker with which to predict the efficacy of nivolumab before treatment is needed. Furthermore, in the multivariate analysis, NLR was not a prognostic factor for PFS. This might have been due to the number of patients in this study, which may have been too small to reveal a significant difference in NLR in the multivariate analysis. Finally, we did not use immune-related response criteria, because this was not a prospective trial and most physicians are not yet familiar with such criteria. We were also unable to examine the expression of PD-L1 before treatment because of the limitation associated with patient insurance policies.

In conclusion, measurement of NLR during nivolumab treatment might be a simple and useful early biomarker in patients with advanced NSCLC. However, its use needs to be evaluated in a larger prospective cohort study.
